# Boltzmann Machines as Generalized Hopfield Networks: A Review of Recent Results and Outlooks

**DOI:** 10.3390/e23010034

**Published:** 2020-12-29

**Authors:** Chiara Marullo, Elena Agliari

**Affiliations:** Dipartimento di Matematica “Guido Castelnuovo”, Sapienza Università di Roma, 00185 Roma, Italy; chiara.marullo@uniroma1.it

**Keywords:** boltzmann machine, hopfield model, statistical mechanics of disordered systems

## Abstract

The Hopfield model and the Boltzmann machine are among the most popular examples of neural networks. The latter, widely used for classification and feature detection, is able to efficiently learn a generative model from observed data and constitutes the benchmark for statistical learning. The former, designed to mimic the retrieval phase of an artificial associative memory lays in between two paradigmatic statistical mechanics models, namely the Curie-Weiss and the Sherrington-Kirkpatrick, which are recovered as the limiting cases of, respectively, one and many stored memories. Interestingly, the Boltzmann machine and the Hopfield network, if considered to be two cognitive processes (learning and information retrieval), are nothing more than two sides of the same coin. In fact, it is possible to exactly map the one into the other. We will inspect such an equivalence retracing the most representative steps of the research in this field.

## 1. Introduction

The two pillars of the cognitive process are the abilities to learn and to retrieve information: one is useless without the other, because there is no reason we should gather information if there is no way to recall it, and we cannot recover notions if we have not previously learnt them. These two aspects of human cognition have been naturally and successfully implemented into machines. The Hopfield network is an artificial neural network which constitutes the theoretical prototype for a wide class of associative memory models; it is defined assuming specific mathematical and physical properties which are more or less aligned to the biological reality. Boltzmann machines are instead the paradigmatic model for learning and are trained using an iterative process whose objective is to construct a probability distribution which mirrors, as closely as possible, the empirical distribution of the training data. In their simplest form, Boltzmann machines are defined as multi-species networks where the nodes are split into two classes (or layers) called visible and hidden respectively: the former codifies the input and the output of the machine, the letter builds up an internal image.

A crucial point is that, under suitable hypothesis, the thermodynamics of the Hopfield model is formally the same as that of the Boltzmann machine. This equivalence has several implications and, in particular, it suggests that the conditions under which the former is able to retrieve can be related to the conditions under which the latter is able to identify features in the input data. Therefore, from both an intuitive and a formal point of view, learning and retrieval are not two independent operations, but rather two complementary aspects of cognition and a large number of results connect these two aspects. The aim of this survey is to provide a general overview of the most important and recent works on this topic.

The paper is organized as follow: In Section 2 we describe the Hopfield model and the Boltzmann machine and, in Section 3, we link them showing their equivalence both with a probabilistic and statistical mechanical approach. Then, in Section 4, we address the case of diluted neural networks observing how this can lead to “parallel retrieval” skills, and, in Section 5, we further extend the range of embedding topologies by considering non-mean-field architectures. Next, in Section 6, we explore the case of dense neural networks, taking into account different types of “synaptic noise” and studying their influence on learning and retrieval of information. Finally, in Section 7, we present some recent works whose goal is to improve the critical capacity of networks.

## 2. Definition of Hopfield Model and Boltzmann Machine

The Hopfield model is the key model for retrieval: its goal is to recognize and reconstruct input vectors that have been previously learned and are now presented in a noisy or incomplete version [1,2]. The model is then composed of a set of *N* neurons $\sigma_{i}\in \left\{-1,+1\right\}$ for i=1,…N and a set of *P* memories, also referred to as patterns, which can be codified in terms of *P* binary vectors of length *N*. More precisely, the *i*-th entry of the μ-th pattern is denoted as $\xi_{i}^{\mu }$ for i=1,…,N and μ=1,…,P, and it is taken as a Rademacher random variable, namely $P(\xi_{i}^{\mu }=\pm 1)=1/2$ ∀i,μ. This model can be seen as a mean-field spin-glass where the interaction matrix *J* is constructed in a special form so that the stored patterns are ground states for the Hamiltonian (vide infra). The neurons evolve according to the dynamic rule

(1) $$\begin{array}{cc} \; & \sigma_{i}\left(t+1\right)=\mathrm{sgn}\left(\sum_{k\neq i}^{N}J_{ik}\sigma_{k}\left(t\right)+z_{i}T+\vartheta_{i}\right), \end{array}$$

where $T\in R^{+}$, $z_{i}$ are i.i.d centered random variables and $\vartheta_{i}$ is the firing threshold of the neurons. Under some conditions (e.g., symmetry of the coupling matrix *J*), we obtain a system that converges to the following equilibrium measure

(2) $$\mu^{\left(H\right)}\left(\sigma ;\xi \right)=\frac{1}{Z^{\left(H\right)}\left(\xi \right)}e^{-\beta H_{N}^{\left(H\right)}\left(\sigma ,\xi \right)},$$

where $Z^{\left(H\right)}\left(\xi \right):=\sum_{\sigma }e^{-\beta H_{N}^{\left(H\right)}\left(\sigma ,\xi \right)}$ is the normalization factor, also referred to as partition function, β:=1/T, and $H_{N}^{\left(H\right)}$ is the Hamiltonian of the system reading as

(3) $$H_{N}^{\left(H\right)}\left(\sigma ,\xi \right)=-\frac{1}{2}\sum_{i,j=1}^{N}J_{ij}\sigma_{i}\sigma_{j}+\sum_{i=1}^{N}\vartheta_{i}\sigma_{i}.$$

As anticipated, the interaction matrix *J* is constructed in such a way that, as long as the noise level is not too large, the dynamics (1) ensures retrieval, namely relaxation towards configurations $\sigma =\xi^{\mu }$; the particular μ-th memory selected depends on the initial configuration σ(t=0) interpreted as the input given to the machine [1,2]. In this context noise alludes to both the degree of randomness in the state of a neuron (this is tuned by *T* and also referred to as fast noise) and the interference among patterns (this is tuned by $\alpha_{N}:=P/N$ and also referred to as slow noise). A possible choice for couplings is

(4) $$J_{ij}:=\frac{1}{N}\sum_{\mu =1}^{P}\xi_{i}^{\mu }\xi_{j}^{\mu }$$

which ensures that the states $\sigma =\xi^{\mu }$ for μ=1,…,P are fixed points for the noiseless dynamics (T=0) and, as long as *T* and *P* are relatively small, the measure (2) concentrates on retrieval neural configurations. Further, the formula in (4) implements Hebb’s learning rule, which states that the change in synaptic strength acting between two neurons is proportional to the correlation between the activity of the presynaptic neuron and the postsynaptic one [3].

The order parameter used to quantify retrieval is the so-called Mattis magnetization, defined as

(5) $$m_{\mu }:=\frac{1}{N}\sum_{i=1}^{N}\sigma_{i}\xi_{i}^{\mu },\;\;\mu =1,\ldots ,P.$$

Please note that as pattern entries are Rademacher random variables, patterns are orthogonal (at least as N→∞) and therefore retrieval is sequential, i.e., if $m_{\mu }\approx 1$ for some μ, then $m_{\nu }\approx 0$ for ν≠μ. Otherwise stated, only one pattern per time can be correctly retrieved; mixtures of patterns such as $\sigma_{i}=\mathrm{sgn}\left(\xi_{i}^{\mu }+\xi_{i}^{\nu }+\xi_{i}^{\rho }\right)$ for i=1,…,N, can, under suitable conditions for *T* and *P*, correspond to attractive points and yield to simultaneously non-null magnetizations ($m_{\mu },m_{\nu },m_{\rho }$), but they are considered as spurious and to be avoided as they may inhabit the dynamics of the network toward a correct retrieval [1].

Boltzmann machines are instead the paradigmatic model for learning (see e.g., [2,4,5]). We can imagine this machine as a network composed of N+P+K neurons that are split into an input layer (of *N* neurons), a hidden layer (of *P* neurons), and an output layer (of *K* neurons), and whose connectivity is described by a symmetric interaction matrix $W_{ij}$, with i,j∈{1,…,N+P+K}. Notice that coupling symmetry makes the network recurrent and an absent interaction simply corresponds to $W_{ij}=0$. Potentially, interactions can involve neurons belonging to different layers as well as neurons belonging to the same layer, yet the matrix *W* is often taken as sparse retaining, as a minimum requirement, nonzero interactions between the input layer and the hidden layer, and between the hidden layer and the output layer, in order to ensure that input signals can at least reach the output side. Here, we will focus on this minimal structure, also referred to as restricted Boltzmann machine (RBM).

The Hamiltonian (or cost function in a machine learning jargon) for the Boltzmann machine reads as

(6) $$H_{N}^{\left(B\right)}\left(s,W\right)=-\frac{1}{2}\sum_{i,j}W_{ij}s_{i}s_{j}-\sum_{i}s_{i}\vartheta_{i},$$

where *s* is the combined state of neurons in all three layers and the parameter $\vartheta_{i}$ determines the firing threshold of the *i*-th neuron. Also for this model, by properly letting the neurons evolve, one can find an equilibrium measure

(7) $$\mu^{\left(B\right)}\left(s;W\right)=\frac{1}{Z^{\left(B\right)}\left(W\right)}e^{-\beta H_{N}^{\left(B\right)}\left(s,W\right)}$$

where $Z^{\left(B\right)}\left(W\right):=\sum_{s}e^{-\beta H_{N}^{\left(B\right)}\left(s,W\right)}$ is the related partition function.

This machine undergoes a training process along which the parameters *W* and ϑ are iteratively updated in such a way that the equilibrium distribution $\mu^{\left(B\right)}\left(s;W\right)$ restricted to visible neurons mirrors an unknown, target distribution. The training is accomplished by exploiting a training data set which is made of a sample of data drawn from the target distribution. Therefore, the system reaches its task when its equilibrium input-output probability distribution satisfactorily mimics the (empirical) target distribution. By minimizing the Kullback-Leibler distance between these two distributions one gets the following learning rule [2]

(8) $$\begin{array}{cc} \; & \Delta W_{ij}=\frac{\varepsilon \beta }{\ln2}\left({\langle s_{i}s_{j}\rangle }_{+}-{\langle s_{i}s_{j}\rangle }_{-}\right)\\ \; & \Delta \vartheta_{i}=\frac{\varepsilon \beta }{\ln2}\left({\langle s_{i}\rangle }_{+}-{\langle s_{i}\rangle }_{-}\right), \end{array}$$

where 0<ε≪1 is the learning rate and averages indicated with ‘+’ are those where the system is only allowed to change the states of hidden neurons, while averages indicated with ‘−’, describe a system where only hidden and output neurons are free to evolve (Such a training makes the machine able to associate to a given input a certain output in agreement with the unknown target distribution. An analogous learning rule can be obtained where averages indicated with ‘−’, describe a system where all neurons are free to evolve. That kind of training makes the machine able to generate input-output examples as if they were drawn from the target distribution.). The meaning of these rules is: the machine tries to learn the statistical structure of the data it has been exposed to, by reproducing the lowest order correlations functions. Clearly, as long as we deal with Gaussian-like data, one-point and two-point correlations functions (accounting for mean and variances in the available data) suffice; this motivates our focus on the equivalence for shallow networks. Different algorithms have been developed for an efficient training, among them we mention the celebrated Hinton’s contrastive divergence [5,6,7].

To simplify the treatment, in the following we will focus on systems where the firing threshold (i.e., the external field in the statistical-mechanics perspective) is set equal to zero, that is $\vartheta_{i}=0$, for any *i*.

## 3. Formal Equivalence between Hopfield Model and Boltzmann Machine

Being two complementary aspects of cognition, learning and retrieval must be connected. It is then natural to ask how the information captured during training by a learning machine and codified by a set of training parameters (e.g., *W* for the Boltzmann machine) can be related to the information stored by an associative memory through a set of prescribed parameters (e.g., *J* for the Hopfield model).

To see this, for the sake of simplicity, we will consider the simplest architecture for the Boltzmann machine, namely a two-layer network, i.e., as a bipartite spin-glass. We use the symbol $\sigma_{i}$, i∈{1,…,N} for neurons in the visible layer, $z_{\mu }$, μ∈{1,…,P} for those in the hidden layer and $W_{i\mu }$ for the couplings between the neuron *i* in one layer and the neuron μ in the other layer. Further, we will focus on the case of hybrid two-layer restricted Boltzmann Machine (HRBM) in which the activity of the neurons in the visible layer is Boolean ($\sigma_{i}\in \left\{-1,+1\right\}$, ∀i=1,…,N), while in the hidden layer is continuous and Gaussian distributed ($z_{\mu }∼N\left(0,\beta^{-1}\right)$, ∀μ=1,…,P). We can then write the Hamiltonian for the HRBM as

(9) $$H_{N}^{\left(B\right)}\left(\sigma ,z,W\right)=-\frac{1}{\sqrt{N}}\sum_{i=1}^{N}\sum_{\mu =1}^{P}W_{i\mu }\sigma_{i}z_{\mu }.$$

The statistical mechanics investigation of this system is based on the related quenched pressure defined as

(10) $$F_{N}^{\left(B\right)}\left(\beta \right):=\frac{1}{N}E\log Z_{N}^{\left(B\right)}\left(W\right)=\frac{1}{N}E\log\sum_{\left\{\sigma \right\}}\int \prod_{\mu =1}^{P}d\mu \left(z_{\mu }\right)\exp\left(\frac{\beta }{\sqrt{N}}\sum_{i=1}^{N}\sum_{\mu =1}^{P}W_{i\mu }\sigma_{i}z_{\mu }\right),$$

where $d\mu (z_{\mu })$ is the Gaussian measure with variance $\beta^{-1}$. Since there are no connections within each party, the sums can be factorized and carrying out the integration over *z* we get

(11) $$F_{N}^{\left(B\right)}\left(\beta \right)=\frac{1}{N}E\log\sum_{\left\{\sigma \right\}}\exp\left(\frac{\beta }{2N}\sum_{i,j=1}^{N}\sum_{\mu =1}^{P}W_{i\mu }W_{j\mu }\sigma_{i}\sigma_{j}\right).$$

**Figure 1:**
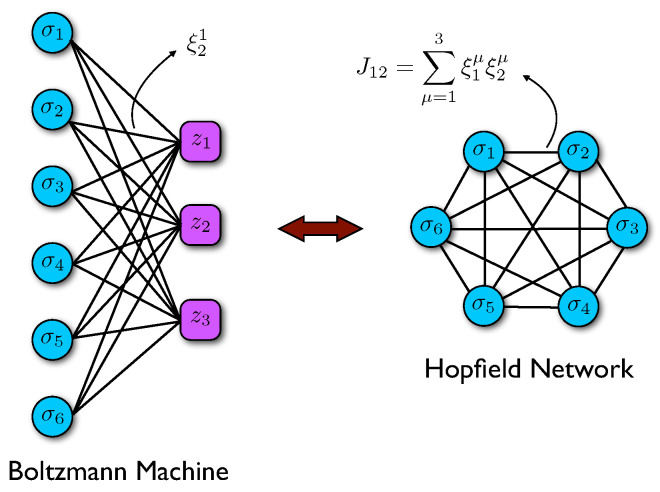
Schematic representation of the equivalence between a two-layer HRBM (**left**) and an Hopfield network (**right**). Please note that the size of the visible layer (here N=6) and of the hidden layer (here P=3) in the former correspond, respectively, to the size and to the number of stored patterns in the latter.

It is immediate to check the equivalence with the free energy of the Hopfield model, that is

(12) $$F_{N}^{\left(H\right)}\left(\beta \right):=\frac{1}{N}E\log Z_{N}^{\left(H\right)}\left(\xi \right)=\frac{1}{N}E\log\sum_{\left\{\sigma \right\}}\exp\left(\frac{\beta }{2N}\sum_{i,j=1}^{N}\sum_{\mu =1}^{P}\xi_{i}^{\mu }\xi_{j}^{\mu }\sigma_{i}\sigma_{j}\right)$$

as long as we identify $W_{i\mu }=\xi_{i}^{\mu }$, ∀μ,i, see Figure 1.

It is possible to recover the equivalence between the two models also from a probabilistic point of view (see Appendix A for more details). In fact, by studying the dynamics of the variables $\sigma_{i}$ in the visible layer and of the variables $z_{\mu }$ in the hidden layer one can write the joint distribution of the variables involved as

(13) $$P\left(\sigma ,z\right)\propto \exp\left(-\frac{\beta }{2}\sum_{\mu =1}^{P}z_{\mu }^{2}+\beta \sum_{i,\mu =1}^{N,P}\sigma_{i}W_{i\mu }z_{\mu }\right)$$

and the marginal distribution describing the statistics of visible neurons as

(14) $$P\left(\sigma \right)\propto \exp\left[-\frac{\beta }{2}\sum_{i,j=1}^{N}\left(\sum_{\mu =1}^{P}W_{i\mu }W_{j\mu }\right)\sigma_{i}\sigma_{j}\right].$$

This probability distribution is equal to the equilibrium distribution of a Hopfield network (see Equations (2) and (3)), where the synaptic weights $J_{ij}$ of the Hopfield network are given by the expression in round brackets just in analogy with (4).

Therefore, the stored patterns of the Hopfield model correspond to the weights of the trained HRBM, and the number of patterns corresponds to the number *P* of hidden units. In other words, retrieval in the Hopfield network corresponds to the case in which the HRBM learns to reproduce a specific pattern of neural activation. Having in mind this formal analogy we can from now on use $\xi_{i}^{\mu }$ to denote the trained weights in the HRBM.

Once this connection is established, what is known for the Hopfield model can be in principle translated to the study of the retrieval capability of a trained RBM. As we will see in the next sections, the formal equivalence between Boltzmann machines and Hopfield networks is interesting not only from a theoretical perspective, but also provides new ideas and approaches to tackle existing problems as well as inspiration for opening new strands of investigation (see e.g., [4,8,9,10]). For instance, we recall that the Hopfield network capacity $\alpha_{c}$, namely the maximum number of patterns per neuron that can be correctly retrieved in the thermodynamic limit is approximately 0.14 [1]. If the number of patterns exceeds this limit, the network is not able to retrieve any of them. On the other hand, if the RBM has too large a number *P* of hidden variables, this provokes over-fitting and the RBM is not able to reproduce the statistics of the observed system. The correspondence between the Hopfield network and the Boltzmann machine is perfectly consistent with this evidence.

Before proceeding, it is worth stressing that the analogy between the Hopfield model and the two-layer HRBM can be extended to more general settings including, for instance, generalized models where the nature of neurons can span from binary to continuous (see [11,12] and the next section), models embedded in complex topologies (see [13,14,15,16,17,18] and Section 5, Section 6 and Section 7), three-layers RBM [19], and non-restricted hybrid BMs.

## 4. On the Nature of Neurons

In Section 3 we focused on a hybrid machine where weights are binary variables while, typically, in machine learning models, the weights are real variables. This evidence prompted the study of general models where the nature of spins ranges continuously between the Boolean and the real Gaussian limits [11,12]. To this aim, one can introduce a joint probability density P(σ,z|ξ) for a generalized RBM, where ξ represents the set of parameters (i.e., interlayer couplings), σ represents the visible units (i.e., the spin state on one layer), and *z* represents the hidden units (i.e., the spin state on the other layer) as

(15) $$P\left(\sigma ,z;\xi \right)=\frac{P_{\sigma }\left(\sigma \right)P_{z}\left(z\right)\exp\left(\sum_{i=1}^{N}\sum_{\mu =1}^{P}\xi_{i}^{\mu }\sigma_{i}z_{\mu }\right)}{Z(\xi )},$$

being $P_{\sigma }$ and $P_{z}$ the spin distributions, which can be interpreted as generic priors, and Z(ξ) is the partition function. The marginal distribution on the visible layer turns out to be

(16) $$P\left(\sigma ;\xi \right)=\frac{1}{Z(\xi )}P_{\sigma }\left(\sigma \right)\exp\left[\sum_{\mu =1}^{P}u\left(\sum_{i=1}^{N}\xi_{i}^{\mu }\sigma_{i}\right)\right]$$

with $u\left(x\right)=\log E_{z}e^{xz}$ the cumulant generating function of hidden unit prior. Notice that P(σ,z;ξ) can be interpreted as the equilibrium distribution of a generalized RBM, while P(σ;ξ) can be interpreted as the equilibrium distribution of a generalized Hopfield model. Given this framework, we distinguish a learning process, meant to find an optimal set of parameters ξ by adapting the marginal distribution P(σ|ξ) to the data (e.g., via likelihood maximization) [7,20,21,22,23], and an inference process where, having learnt the optimal parameters, the hidden units are selectively activated by the data characteristics through P(z|σ,ξ). The reliability of learning and inference depends on the dataset but also on the choice of the generative model used.

As anticipated, the priors of the visible and hidden units and weights can be generic and, denoting with η∈{ξ,σ,z} the generic random variable, we can choose

(17) $$P_{\eta }\left(\eta ;\Omega_{\eta }\right)\propto \sum_{\varepsilon =\pm 1}\exp\left(\frac{-{\left(\eta -\sqrt{1-\Omega_{\eta }}\varepsilon \right)}^{2}}{2\Omega_{\eta }}\right),$$

where $\Omega_{\eta }\in \left[0,1\right]$ is the interpolation parameter associated with the random variable η. Clearly, when $\Omega_{\eta }\to 0$, $P(\eta ;\Omega_{\eta })$ converges to a binary distribution while, for $\Omega_{\eta }\to 1$ the distribution will be Gaussian. Here both the patterns and the spins are drawn from such a distribution, hence $\xi_{i}^{\mu }∼P_{\xi }\left(\xi ;\Omega_{\xi }\right)$, $\sigma_{i}∼P_{\sigma }\left(\sigma ;\Omega_{\sigma }\right)$ and $z_{\mu }∼P_{z}\left(z;\Omega_{z}\right)$ ∀i=1,…,N and μ=1,…,P.

**Figure 2:**
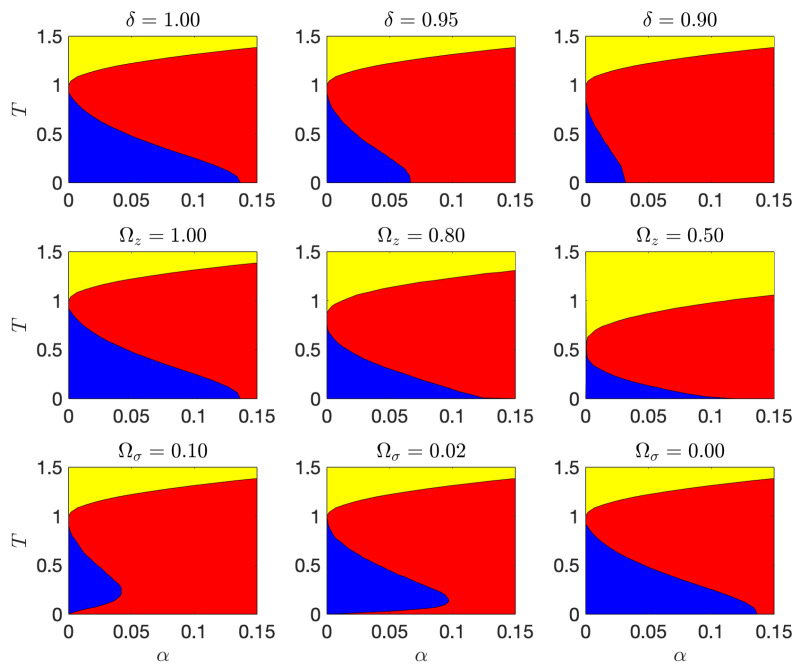
Phase diagram of a generalized RBM for varying pattern, hidden and visible unit priors as found in [12]. In all plots, the yellow region represents the ergodic phase, the red region represents the spin-glass phase, and the blue region represents the retrieval phase. First line: the visible units are Boolean and $\Omega_{z}=1$. In this case the retrieval region approaches the line α=0 and $T\in [0,\Omega_{z}]$ as δ→0. Second line: the visible variables are Boolean and δ=1. The retrieval region approaches the line T=0 and $\alpha \in [0,\alpha_{c}\left(\delta \right)]$ as $\Omega_{z}\to 0$. Third line: δ=1, $\Omega_{z}=1$ and the soft visible units are regularized with a spherical constraint. As $\Omega_{\sigma }\to 0$ the retrieval region approaches low load values.

Introducing $\delta =\sqrt{1-\Omega_{\xi }}$ and rescaling the variables $\xi_{i}^{\mu }\mapsto \sqrt{\beta /N}\xi_{i}^{\mu }$, where β is the inverse temperature, it was possible to apply statistical-mechanics (under replica symmetry assumption) to study the asymptotic phase diagram of the model (15) in the limit of large *N* as a function of $\alpha_{N}$ and *T* [11,12]. In particular, it can be seen that for large values of *T* a paramagnetic phase occurs while, as *T* decreases, a phase transition towards the spin-glass regime, characterized by frozen disorder, is observed. Finally, when *T* is further reduced, we find a retrieval region where the visible layer is significantly related to a particular pattern, which is recovered. Moreover, the retrieval region shrinks as δ and $\Omega_{z}$ decrease, while it gradually increases for smaller and smaller values of $\Omega_{\sigma }$. These facts are summarized in Figure 2.

## 5. Pattern Dilution and Multitasking Capacities

Since its introduction in 1982 [24], the Hopfield model has been intensively investigated and many variations on theme have been introduced. A natural one, inspired by the relative sparseness of biological neural networks, consists of introducing dilution in the embedding structure. For instance, Sompolinsky showed that the random dilution of synapses lead to a decrease in the maximum capacity of the network proportional to the percentage of dilution [25]. Later on, Coolen et al. [26] studied symmetrically diluted attractor neural networks with finite connectivity, where the average number *c* of bonds per neuron is finite, independent of the system size. Using finite connectivity spin-glass replica techniques it was possible to obtain the phase diagram of the model in the (γ,T) plane for arbitrary finite *c*, where γ=P/c and *P* is the number of stored patterns. This diagram shows three phases (paramagnetic, retrieval and a spin-glass phase) all separated by second-order transitions. Further results on diluted neural networks are due to Derrida et al. (see e.g., [27,28,29]).

The duality described in the previous section suggests another kind of dilution, this time implemented in the links of the Boltzmann machine and therefore on patterns entries in the Hopfield model, which, as shown in [13,14], gives rise to interesting behaviors. In particular, as we are going to review in the following, the system turns out to be able to retrieve several patterns simultaneously without falling in a spurious state, i.e., it is able to perform “parallel processing”.

The introduction of dilution in pattern entries can be formulated as

(18) $$P\left(\xi_{i}^{\mu }\right)=\frac{1-d}{2}\delta_{\xi_{i}^{\mu },-1}+\frac{1-d}{2}\delta_{\xi_{i}^{\mu },+1}+d\;\delta_{\xi_{i}^{\mu },0},$$

where d∈[0,1] is a parameter tuning the degree of dilution. It is evident that here we are removing the hypothesis of full connection for the bipartite network corresponding to the RBM, while, as long as the dilution is not extreme, the corresponding Hopfield model remains embedded in a fully connected network. However, now, since the *P* patterns $\xi^{\mu }$, in the average, contain zeros for a fraction *d* of their length, the retrieval of a pattern does not employ all spins and those corresponding to blank entries can be used to recall other patterns. As found in [13,14] via statistical-mechanica investigations, for T→0 and at a relatively low degree of dilution, one pattern, say $\xi^{1}$, is perfectly retrieved and its related Mattis magnetization (see Equation (5)) is $m_{1}=1-d$, thus a fraction *d* of spins is still available and the most convenient arrangement is the one where they align with one, say $\xi^{2}$, among the remaining patterns in such a way that $m_{2}=d\left(1-d\right)$. By iteration we find that the retrieval of the *k*-th pattern is assessed by

(19) $$m_{k}=d^{k-1}\left(1-d\right).$$

For any fixed and finite *d*, this implies that the number of patterns retrievable at least partially scales at most logarithmically with *N*. This can be thought of as a “parallel low-storage” regime of neural networks. For large values of dilution, however, none of the magnetizations is sufficiently large to produce a field $\xi_{i}^{\mu }m_{\mu }$ that aligns all the related spins. The system then falls into a spurious state in which all patterns are only partially recovered. Whenever the equilibrium configuration of the system corresponds to the perfect retrieval of at least one pattern, we refer to “multitasking capabilities” or “parallel retrieval”. Therefore, when the stored information is partially blank, the resulting associative network is not only still able to perform the retrieval, but it can actually retrieve several patterns at the same time without falling into spurious states; this autonomous parallel processing can be exploited in various application contexts [13,14,30].

**Figure 3:**
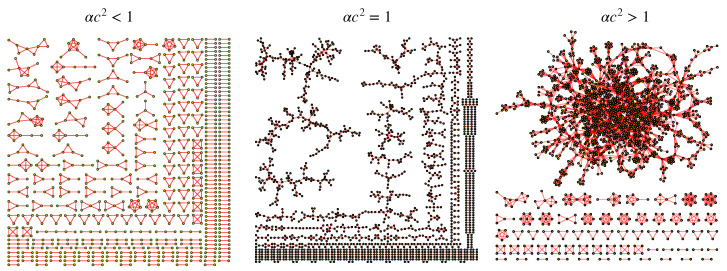
Examples of typical graphs obtained for different values of *c* and α; in any case the size is N=4000. **Left**: a picture corresponding to the under-percolated regime (α=0.4,c=1). **Middle**: a picture corresponding to the percolation threshold (α=c=1). **Right**: a picture corresponding to the over-percolated regime (α=0.1,c=5). Notice that isolated nodes are not depicted.

The case of strongly diluted networks has been treated in [14]. In particular, the dilution is varied according to the scaling $d=1-\frac{c}{N}$ and, as *c* is varied, one can study the effects on the structures embedding the Hopfield model and the Boltzmann machine. It is interesting to observe that the point where $\alpha c^{2}=1$ defines the percolation threshold for the bipartite graph: for $c<1/\sqrt{\alpha }$ the graph appears fragmented in many components while for $c>1/\sqrt{\alpha }$ a giant component emerges (see Figure 3). A similar behavior can also be found in the monopartite graph resulting from marginalization, where the percolation threshold becomes αc=1. We underline that disconnected components promote simultaneous recall of multiple patterns while the presence of a giant component hinders parallel processing ability.

These insights, obtained from a topological perspectives, can be recovered also from a statistical mechanics investigation employing techniques suitable for finitely connected spin systems. Indeed, one can find a critical surface $T_{c}\left(\alpha ,c\right)$ that separates two distinct phases: when $T<T_{c}\left(\alpha ,c\right)$ interference among patterns prevails and parallel retrieval is compromised, while when $T>T_{c}\left(\alpha ,c\right)$, the system behaves as a large ensemble of independent finite-sized neural networks, each storing a single undiluted pattern, in such a way that the only source of noise are the thermal fluctuations within each subsystem (for high temperatures, each subsystem behaves like a paramagnet while, for low temperature values, each of them can retrieve a particular pattern or its inverse thus making the network able to perform a simultaneous retrieval). The critical temperature becomes zero when $\alpha c^{2}=1$, i.e., $T_{c}\left(\alpha ,1/\sqrt{\alpha }\right)=0,\forall \alpha \geq 0$, so for $\alpha c^{2}<1$ no transition at finite temperature away from this phase is possible.

## 6. Hopfield Networks on Complex Topologies

As can be noted from the previous sections, statistical mechanics is a powerful technique for understanding neural networks [1,2,15,31,32], and the mean-field assumption is predominant in most works in this field. Basically, this kind of approximation lies in the assumption that each spin/neuron in a network dialogues with all other spins/neurons with the same strength and this corresponds to a system embedded in a fully connected topology.

One of the simplest non-trivial topologies is constituted by the Erdös-Rényi graph which can be obtained starting from a complete graph and deleting its links with a finite probability d∈[0,1]. As a result, the normalized number of neighbors for each node is a random variable with expectation 1−d. This situation, preserving the homogeneity of the structure and an extensive coordination number, can still be treated as a mean-field model and the results obtained are qualitatively analogous to those of complete graphs. However, real networks often exhibits features such as large clustering coefficients, correlated degrees, small-world properties, and heavy-tailed degree distributions which make the Erdös-Rényi graph too poor a model.

Recently, progress has been made towards the study of more realistic systems by merging statistical mechanics [33,34,35] and graph theory [36,37,38]. In particular, mathematical methods have been developed in order to deal with spin systems incorporated in random graphs where the ideal full homogeneity among spins is lost (see e.g., [13,14,39,40]).

The (strongly) diluted neural networks treated in the previous section could be faced still relying on a Poisson distribution for the degree of the constituting units. The case of highly inhomogeneous topology has been treated in [15]. There, a two-layer architecture is considered where the spins exhibit general degree distributions, covering even scale-free RBMs. In particular, by using cavity methods (i.e., belief-propagation) it was possible to investigate finitely connected bipartite spin-glasses with arbitrary structure and some degree of asymmetry in the link distribution, as well as their thermodynamically equivalent associative networks with diluted patterns where the dilution is modulate by the parameter *c* (we recall that $P(\xi_{i}^{\mu }=0)=1-c/N$, with $c=O(N^{0})$). In fact, it was possible to identify a transition surface separating the region of the (α,β,c)-space where the network is able to perform an extended and parallel retrieval, from the region where pattern interference affects the performance of the network as a parallel processor. Interestingly, the region where parallel retrieval occurs is larger for degree distributions with smaller variance and the optimal situation arises when all patterns have exactly the same number of non-zero entries.

In addition, one can introduce a bias in the pattern entry distribution that may favour positive or negative items; a biased distribution of sparse patterns entries can produce a macroscopic magnetization of the network and narrow the region of the parameter space where no cross-talk among patterns occurs.

## 7. Dense Networks

As mentioned before, the Hopfield network is able to retrieve a number of patterns P=αN with $\alpha \leq \alpha_{c}\approx 0.14$ [1,24]. This threshold is far below the upper bound $\alpha_{\mathrm{ub}}=1$ found by Gardner via replica trick for symmetric networks [41] and, over the years, many efforts were spent to improve the critical capacity of the Hopfield network [27,42,43,44,45,46]. Among the possible approaches, we mention the relaxation of the constraint that neurons can interact only pairwisely, hence allowing for interactions among *k*-neurons (k>2), a revision of the Hebbian coupling (4) towards non-local rules, or a revision of the pattern entries so to include some degree of correlation (however, notice that in this case the information content of patterns is reduced). Regarding the former approach it is worth recalling the results obtained by Baldi and Venkatesh [47]: they observed that for an associative *k*-neuron memory built with *N* binary neurons, the largest number of patterns that can be stored scales as $N^{k-1}$.

Hereafter we just focus on these “dense” neural networks [48], generally meant as models embedded in hypergraphs and, in particular, on the Hopfield network displaying interactions among *k*-neurons as well as on the related Boltzmann machine.

A recent result concerning dense neural networks is related to the so-called restricted Sejnowski machine (RSM) [49], that is a three-layer spin-glass with symmetric triplet interactions. As reported in [16], the features learnt via contrastive divergence have a dual representation as patterns in a dense associative memory of order 4 and, by keeping the dense associative network far from the saturation regime, such a system is able to perform pattern recognition far below the standard signal-to-noise threshold. As we will see, this is accomplished exploiting redundancy in the information input to the network.

Let us then consider an RSM with two visible layers and a standard hidden layer in which the first visible layer represents the primary channel and the second visible layer its mirror. The visible layers are digital and made up of *N* binary neurons per layer, denoted with $\sigma \in {\left\{-1,+1\right\}}^{N}$ and $\tau \in {\left\{-1,+1\right\}}^{N}$ respectively, while the hidden layer is analog and made of *P* neurons denoted with *z*, whose states are i.i.d. Gaussians $N∼(0,\beta^{-1})$. The model presents third-order interactions among neurons of different layers but no intra-layer interactions (whence the restriction). Its Hamiltonian $H_{N}^{\left(RSM\right)}$ is given by

(20) $$H_{N}^{\left(RSM\right)}\left(\sigma ,\tau ,z|\xi \right)=-\frac{1}{N^{3/2}}\sum_{i,j\mu =1}^{N,N,P}\xi_{ij}^{\mu }\sigma_{i}\tau_{j}z_{\mu },$$

with i,j=1,…,N and μ=1,…,P. In the thermodynamic limit each layer size diverges such that $\lim_{N\to \infty }P/N=\alpha >0$ and the factor $N^{-3/2}$ keeps the mean value of the Hamiltonian linearly extensive in *N*. The interaction between each triplet of neurons is encoded in the P×N×N tensor ξ whose μ-th element will be written as

(21) $$\xi_{ij}^{\mu }=\xi_{i}^{\mu }\xi_{j}^{\mu }\;\;\;i,j=1,\ldots ,N,$$

where $\xi_{i}^{\mu }\in \left\{-1,+1\right\}$ is meant as the *i*-th entry of the μ-th pattern to be retrieved in the dual dense associative memory, i.e., a bipartite Hopfield model with 4-wise interactions (vide infra). Once a learning rate ε>0 is set, it is possible to obtain the following contrastive-divergence learning rule

(22) $$\Delta \xi_{ij}^{\mu }=\varepsilon \beta \left({\langle \sigma_{i}\tau_{j}z_{\mu }\rangle }_{+}-{\langle \sigma_{i}\tau_{j}z_{\mu }\rangle }_{-}\right)$$

where the subscript “+” means that both visible and mirror layers are set at the data input (i.e., they are clamped), while the subscript “−” means that all neurons in the network are left free to evolve. Writing he partition function associated to the cost function (20) and marginalizing it over the hidden layer one gets

(23) $$Z^{\left(DAM\right)}\left(\xi \right)=\sum_{\sigma ,\tau }\exp\left[-\frac{\beta }{2N^{3}}\sum_{\mu =1}^{P}{\left(\sum_{i,j=1}^{N,N}\xi_{ij}^{\mu }\sigma_{i}\tau_{j}\right)}^{2}\right]=\sum_{\sigma ,\tau }\exp\left(\beta H_{N}^{\left(DAM\right)}\left(\sigma ,\tau |\xi \right)\right)$$

where $H_{N}^{\left(DAM\right)}$ corresponds to a 4-bipartite Hopfield model. Now, we can allow for noise in pattern entries by replacing $\xi_{ij}^{\mu }$ with $\eta_{ij}^{\mu }$ defined as

(24) $$\eta_{ij}^{\mu }:=\xi_{ij}^{\mu }+\sqrt{P}{\tilde{\xi }}_{ij}^{\mu },$$

where the ${\tilde{\xi }}_{ij}^{\mu }$’s are i.i.d. standard Gaussian variables. This kind of noise, being linearly extensive in the network size, might look overwhelming with respect to the signal, and, in fact, it would lead to a retrieval breakdown in pairwise networks, yet, here, checking the stability of retrieval configurations by signal-to-noise analysis or even by statistical mechanics investigations (at the replica symmetry level) it is possible to highlight the existence of a region in the (α,T)-plane where the network can still retrieve patterns (for more details see [16]). The robustness of the network performance stems from the redundancy ensured by the twin visible layers.

The generalization of the duality between the Hopfield model and the Boltzmann machine in case of *k*-neuron interaction suggests the introduction of different types of “synaptic noise” affecting the tensor $J_{i_{1}i_{2}\ldots i_{k}}$ and corresponding to shortcomings that occur at different stages of the cognitive process [16,47,50,51]. In particular, we can outline three possible situations: the case where the patterns supplied during training are noisy, the case where the learning process is impaired by some flaws and the case where storing displays some defects [17].

When the information to be stored is provided with some mistakes we have corrupted patterns given by

(25) $$\eta_{i}^{\mu }=\xi_{i}^{\mu }+\omega {\tilde{\xi }}_{i}^{\mu }$$

where ${\tilde{\xi }}_{i}^{\mu }$ is a standard Gaussian random variable and ω is a real parameter that allows to tune the noise level. In this situation, the signal-noise analysis shows that if ω∼1, no matter how many *P* patterns are stored (up to $P∼N^{k-1}$), retrieval is possible, while if $\omega ∼O(N^{b})$, b>0 it is not possible to handle synaptic noise even if the number of spins is arbitrarily increased.

The second case is the one corresponding to an imperfect learning where the noise affects the (k/2+1)-component tensor

(26) $$\eta_{i_{1}\ldots i_{k/2}}^{\mu }=\xi_{i_{1}}^{\mu }\ldots \xi_{i_{k/2}}^{\mu }+\omega {\tilde{\xi }}_{i_{1}\ldots i_{k/2}}^{\mu }.$$

In this situation retrieval is still possible, but the critical capacity of the network decreases.

Finally, one can consider noise acting directly on the couplings of the associative memory as

(27) $$J_{i_{1}\ldots i_{k}}^{\mu }=\sum_{\mu }\eta_{i_{1}\ldots i_{k}}^{\mu },$$

where $\eta_{i_{1}\ldots i_{k}}^{\mu }=\xi_{i_{1}}^{\mu }\ldots \xi_{i_{k}}^{\mu }+\omega {\tilde{\xi }}_{i_{1}\ldots i_{k}}^{\mu }$. This situation can be associate to shortcomings in the string process. Here, again, an increase of the load results in a decrease of the tolerance.

Thus, depending on how synaptic noise is implemented, the effects on retrieval may vary qualitatively. If the data is provided correctly during learning, synaptic noise can be faced by allowing neurons to interact in relatively large cliques or working in low load regime. On the other hand, if the machine is supplied with pieces of corrupted information, the machine will learn noise as well and, consequently, information can only be reconstructed, independently of the network redundancy, if the original corruption is not divergent.

## 8. Exploration of Boltzmann Machine Capacities

As discussed at the beginning of Section 7, another way to improve the capacity of the network is to revise the Hebbian rule (4). In these regards, it is worth recalling the works by Crick and Mitchinson [52], where they found evidence that mammals during the rapid-eye-movement (REM) sleep phase erase information unintentionally stored during the day, saving memory and avoiding overloading catastrophes. Starting from these results, Hopfield modified his model in order to mimic unlearning mechanisms of irrelevant memories and possibly improving its capacity [53]. His work, along with successive developments (see e.g., [43,44,45,54]), led to the following revision of the Hebbian coupling:

(28) $$J_{ij}=\frac{1}{N}\sum_{\mu ,\nu =1}^{P}\xi_{i}^{\mu }{\left(I+tC\right)}_{\mu ,\nu }^{-1}\xi_{j}^{\nu },$$

where $t\in R^{+}$ is a tunable parameter which can be interpreted as the sleeping time and *C* is the correlation matrix defined as

(29) $$C_{\mu ,\nu }:=\frac{1}{N}\sum_{i=1}^{N}\xi_{i}^{\mu }\xi_{i}^{\nu }.$$

The resulting neural network model was studied by Dotsenko et al. [45] through statistical mechanics and they highlighted that the maximal storage capacity increases as *t* gets larger and larger, approaching the Gardner upper bound $\alpha_{\mathrm{ub}}=1$. The limit of this approach resides in the fact that, in the large *t* limit (for which the storage capacity is maximum), the coupling matrix identically vanishes. This leads to an unlearning of pure memories but even to a progressive destruction of stored information. In fact, it can be seen that even though the recovery region is stretched to higher values in α than the standard Hopfield model, it is still confined to smaller and smaller values of fast noise (which eventually disappears when t→∞).

In recent works (28) is slightly revised as [55,56]

(30) $$J_{ij}=\sum_{\mu ,\nu =1}^{P}\xi_{i}^{\mu }\xi_{j}^{\nu }{\left(\frac{1+t}{I+tC}\right)}_{\mu ,\nu },$$

which, beyond the REM sleep, also accounts for the slow wave (SW) sleep; the former produces the removal of unnecessary memories, the latter the consolidation of important ones. The resulting model, referred to as reinforcement and removal (RR) model, extends the unlearning approaches by simultaneously doing remotion of spurious states and reinforcement of pure ones, providing extra stability of these states, finally resulting in a sensibly enlarged and more robust retrieval region.

**Figure 4:**
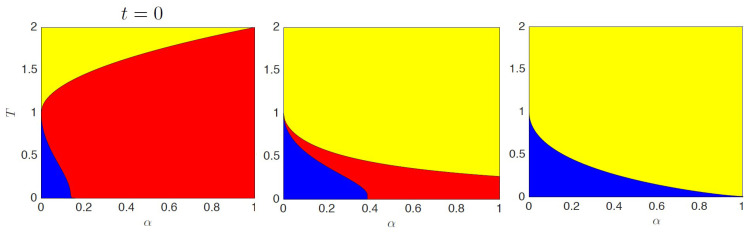
The phase diagram is depicted for different choices of *t*, namely, from left to right, t=0,1,1000. Notice that, as *t* grows, the retrieval region (blue) and the ergodic region (yellow) become larger and larger while the spin-glass region (red) shrinks up to collapse as t→∞. A change in the concavity of the critical line separating the ergodic region and the spin-glass region is also observed.

Let us now introduce the model as follow: consider a network composed by *N* neurons ${\left\{\sigma_{i}\right\}}_{i=1,\ldots ,N}$, with $\sigma_{i}\in \left\{-1,+1\right\}$ ∀i=1,…N, and *P* Boolean patterns $\xi^{\mu }$, with μ=1,…,P. For each sleeping time $t\in R^{+}$, the reinforcement and removal model is described by the Hamiltonian

(31) $$H_{N}^{\left(RR\right)}\left(\sigma ,\xi ,t\right)=-\frac{1}{2N}\sum_{i,j=1}^{N}\sum_{\mu ,\nu =1}^{P}\xi_{i}^{\mu }\xi_{j}^{\nu }{\left(\frac{1+t}{I+tC}\right)}_{\mu ,\nu }\sigma_{i}\sigma_{j}$$

where, as standard, the entries of the *P* patterns are Rademacher random variables. Remarkably, while sleeping, both reinforcement and removal are performed. In fact, in the generalized kernel appearing in (31), the term ${\left(1+tC\right)}^{-1}$ yields to the remotion of unwanted mixture states, while the term 1+t reinforces the memories. It is then possible to obtain, by using the standard variational principle, set of self-consistent equations of the model from which it is possible to study the evolution of the phase diagram in function of the sleep time *t* (Figure 4). We can see that, without sleeping, ergodicity breaks as predicted by Amit, Gutfreund and Sompolinsky [1,43]. As one starts to sleep, the spin-glass region starts to reduce its size until it collapses for large *t*-values. At the end, the phase diagram shows only the retrieval and the ergodic phases (Figure 4).

Using β as a parameter tuning the level of fast noise in the network, the partition function of the RR model can be represented in Gaussian integral form as

(32) $$\begin{array}{cc} Z_{N,P}^{\left(RR\right)}\left(\sigma ,\xi ,t\right)= & \sum_{\sigma }\int \left(\prod_{\mu =1}^{P}d\mu \left(z_{\mu }\right)\right)\left(\prod_{i=1}^{N}d\mu \left(\phi_{i}\right)\right)\cdot \\ & \exp\left(\sqrt{\frac{\beta (t+1)}{N}}\sum_{\mu ,i=1}^{P,N}z_{\mu }\xi_{i}^{\mu }\sigma_{i}+i\sqrt{\frac{t}{N}}\sum_{\mu ,i=1}^{P,N}z_{\mu }\xi_{i}^{\mu }\phi_{i}\right) \end{array}$$

where $d\mu (z_{\mu })$ and $d\mu (\phi_{i})$ are the standard Gaussian measures. From this relation it is possible to see that the partition function of the RR model is equivalent to the partition function of a tripartite tripartite spin-glass in which the first layer is made by a set of Boolean neurons ${\left\{\sigma_{i}\right\}}_{i=1,\ldots ,N}$, the hidden layer is made of real neurons $z_{\mu }∼N\left(0,1\right)$, ∀μ=1,…,P and the last layer is made of a set of imaginary neurons with magnitude ${\left\{\phi_{i}\right\}}_{i=1,\ldots ,N}$, being $\phi_{i}∼N\left(0,1\right)$, ∀i=1,…,N.

In this way, we obtained a Boltzmann machine where we can set the number of hidden variable up to P=N. This allows having many degrees of freedom, i.e., many coefficients that allow to represent reality without falling into the overfitting regime [55].

## 9. Conclusions

This article provides an overview on the two key models of learning and retrieval—the Hopfield model and the Boltzmann machine—focusing on their formal thermodynamic equivalence. Beyond presenting a proof for such an equivalence, we discussed on how this connection can be exploited to obtain theoretical insights and a deeper comprehension of the models as a whole, and can as well provide the inspiration for efficient algorithms and challenging applications. In particular, we described the effects of different kinds of dilution in these networks, possibly yielding to parallel retrieval of stored memories; the properties of dense neural networks and their robustness with respect to different kinds of noise; and revisions in the way couplings among neurons are defined so to obtain improvements in the network performance. We hope that this review will not only provide a survey on the current state of the art but will also stimulate new ideas, investigation methods and results.

## Appendix A. Statistical Equivalence of Rbm and Hopfield Networks

In this section, we will study in more detail the statistical equivalence between a hybrid two-layer Boltzmann Machine and the Hopfield network. As already mentioned in Section 3 we indicate with $\sigma_{i}\in \left\{-1,+1\right\}$ for i=1,…,N the neurons in the visible layer, with $z_{\mu }∼N\left(0,\beta^{-1}\right)$ for μ=1,…,P the variables in the hidden layer and with $\xi_{i}^{\mu }$ the synaptic connections between the input unit $\sigma_{i}$ and the hidden unit $z_{\mu }$.

Being the visible and hidden variables of the network of different nature their time evolution will be different; digital units will change in discrete steps while analog ones will change continuously in time. In particular, variables $\sigma_{i}$ follows a standard neural dynamics of Glauber type [1] in which for each specific time step every visible unit is updated instantaneously. At every step the probabilities of finding digital units in a specific state depends on the state assumed in that instant the hidden variables *z* and determined by the total fields $\sum_{\mu =1}^{P}W_{\mu i}z_{\mu }$ acting on them. Furthermore, the activity of the $\sigma_{i}$ is independent on other units, and the probability is a logistic function of its input, leading to

(A1) $$P\left(\sigma_{i}|z\right)=\frac{\exp\left(\beta \sigma_{i}\sum_{\mu =1}^{P}W_{i\mu }z_{\mu }\right)}{\exp\left(\beta \sum_{\mu =1}^{P}W_{i\mu }z_{\mu }\right)+\exp\left(-\beta \sum_{\mu =1}^{P}W_{i\mu }z_{\mu }\right)}.$$

The activity in the hidden analog layer, instead, is described by the following stochastic differential equation

(A2) $$\tau \frac{dz_{\mu }}{dt}=-z_{\mu }\left(t\right)+\sum_{i=1}^{N}W_{i\mu }\sigma_{i}+\sqrt{\frac{2\tau }{\beta }}\eta_{\mu }\left(t\right)$$

where η is a white Gaussian noise with zero mean and covariance $\langle \eta_{\mu }\left(t\right)\eta_{\nu }\left(t^{\prime }\right)\rangle =\delta_{\mu \nu }\delta \left(t-t^{\prime }\right)$ [57]. Here the parameter β determines the extent of the fluctuations and the parameter τ gives the timescale of the dynamics. The three terms in the right hand side of (A2) are respectively the leakage, the input signal and the noise source; the equation describes a Ornstein-Uhlembeck diffusion process.

The equilibrium distribution of $z_{\mu }$, for fixed values of σ, is a Gaussian distribution centered around the input signal, that is

(A3) $$P\left(z_{\mu }|\sigma \right)=\sqrt{\frac{\beta }{2\pi }}e^{-\frac{\beta }{2}{\left(z_{\mu }-\sum_{i=1}^{N}W_{i\mu }\sigma_{i}\right)}^{2}}.$$

Deriving this probability distribution, we are tacitly assuming that the activity of Boolean units σ must be constant, while in fact it depends on time. To make both features compatible, we should assume that the timescale of diffusion τ is faster than the update rate of neurons in the visible layer.

Since we have the conditional distributions of both layers at our disposal ((A1) and (A3)), by applying Bayes theorem we can determine their joint distribution, P(σ,z), together with the marginal distributions P(z) and P(σ) by the chain of equalities P(σ,z)=P(z|σ)P(σ)=P(σ|z)P(z). Using the fact that marginal distributions depend on single layer variables, the result for the joint distribution is

(A4) $$P\left(\sigma ,z\right)\propto \exp\left(-\frac{\beta }{2}\sum_{\mu =1}^{P}z_{\mu }^{2}+\beta \sum_{i,\mu =1}^{N,P}\sigma_{i}W_{i\mu }z_{\mu }\right).$$

The marginal distribution describing the statistics of visible neurons is equal to

(A5) $$P\left(\sigma \right)\propto \exp\left[-\frac{\beta }{2}\sum_{i,j=1}^{N}\left(\sum_{\mu =1}^{P}W_{i\mu }W_{j\mu }\right)\sigma_{i}\sigma_{j}\right].$$

This probability distribution is equal to the equilibrium distribution of a Hopfield network (see Equations (2) and (3)), where the synaptic weights $J_{ij}$ of the Hopfield network are given by $\sum_{\mu =1}^{P}W_{i\mu }W_{j\mu }$.
